# A Systematic Review of Methods to Incorporate External Evidence into Trial-Based Survival Extrapolations for Health Technology Assessment

**DOI:** 10.1177/0272989X231168618

**Published:** 2023-04-26

**Authors:** Ash Bullement, Matthew D. Stevenson, Gianluca Baio, Gemma E. Shields, Nicholas R. Latimer

**Affiliations:** School of Health and Related Research, University of Sheffield, UK; Delta Hat Limited, Nottingham, UK; School of Health and Related Research, University of Sheffield, UK; Department of Statistical Science, University College London, UK; School of Health Sciences, University of Manchester, UK; School of Health and Related Research, University of Sheffield, UK

**Keywords:** survival analysis, health technology assessment, external evidence, extrapolation systematic review

## Abstract

**Background:**

External evidence is commonly used to inform survival modeling for health technology assessment (HTA). While there are a range of methodological approaches that have been proposed, it is unclear which methods could be used and how they compare.

**Purpose:**

This review aims to identify, describe, and categorize established methods to incorporate external evidence into survival extrapolation for HTA.

**Data Sources:**

Embase, MEDLINE, EconLit, and Web of Science databases were searched to identify published methodological studies, supplemented by hand searching and citation tracking.

**Study Selection:**

Eligible studies were required to present a novel extrapolation approach incorporating external evidence (i.e., data or information) within survival model estimation.

**Data Extraction:**

Studies were classified according to how the external evidence was integrated as a part of model fitting. Information was extracted concerning the model-fitting process, key requirements, assumptions, software, application contexts, and presentation of comparisons with, or validation against, other methods.

**Data Synthesis:**

Across 18 methods identified from 22 studies, themes included use of informative prior(s) (*n* = 5), piecewise (*n* = 7), and general population adjustment (*n* = 9), plus a variety of “other” (*n* = 8) approaches. Most methods were applied in cancer populations (*n* = 13). No studies compared or validated their method against another method that also incorporated external evidence.

**Limitations:**

As only studies with a specific methodological objective were included, methods proposed as part of another study type (e.g., an economic evaluation) were excluded from this review.

**Conclusions:**

Several methods were identified in this review, with common themes based on typical data sources and analytical approaches. Of note, no evidence was found comparing the identified methods to one another, and so an assessment of different methods would be a useful area for further research.

**Highlights**

- This review aims to identify methods that have been used to incorporate external evidence into survival extrapolations, focusing on those that may be used to inform health technology assessment.
- We found a range of different approaches, including piecewise methods, Bayesian methods using informative priors, and general population adjustment methods, as well as a variety of “other” approaches.
- No studies attempted to compare the performance of alternative methods for incorporating external evidence with respect to the accuracy of survival predictions. Further research investigating this would be valuable.

Health technology assessment (HTA) makes use of clinical- and cost-effectiveness evidence for health care interventions to support policy decision making. While not an explicit requirement, contemporary HTA processes usually involve a submission made by the manufacturer of the intervention (i.e., health technology) under assessment, which often requires estimation of overall survival (OS) within a cost-effectiveness analysis for both the intervention (and its comparator[s]), particularly in the context of cancer therapies.^1^ Appropriate estimation of OS over a lifetime horizon is critical to reliably estimate the total costs and outcomes associated with a given treatment strategy.

Typically, estimates of OS for a new intervention and its comparator(s) are based on data collected from a pivotal clinical trial (i.e., the trial intended to serve as the primary basis from which to seek regulatory approval).^2^ However, trial-based extrapolations are subject to a number of limitations, usually related to sample size, data maturity (typically defined based on the duration of follow-up data available^3^), and trial design features, which may confound estimates of OS (such as crossover or subsequent therapy). Clinical trials are designed to evaluate the efficacy of a given intervention (i.e., performance in a controlled setting), whereas HTA is focused on its effectiveness (i.e., performance in a “real-world” setting). To address some of the limitations of trial-based survival estimation for HTA, methods that move away from being based solely on pivotal trial data warrant consideration—a sentiment echoed in a number of previously published studies.^1,4–7^

Jackson et al.^8^ conducted a review of methods used when extrapolating survival from randomized trials using external data (i.e., data collected outside of the pivotal clinical trial). While this review provides a helpful description of methods that have been previously proposed in the literature to incorporate external data into survival extrapolations, the review was not systematic and a number of more recent studies have since been published. In addition, some methods may rely on external information, as opposed to external data, for example, integration of clinical expert opinion. The review by Jackson et al. was concerned only with studies that made use of external data, and so there may be other methods that were not discussed that adopt a broader view of external evidence.

The purpose of this review was to systematically identify methods for estimating OS that incorporate an element of external evidence (i.e., data or information) within the model-fitting process. To our knowledge, no comprehensive systematic review of such methods has been conducted, with the most recent nonsystematic review being that of Jackson et al.^8^ Furthermore, we expand the scope of the review by Jackson et al. by considering methods that leverage external information sources. Following identification of such methods, we aim to describe and categorize each of the methods to provide an overview of current approaches to make use of external evidence within survival estimation and identify areas to focus future research efforts.

## Methods

### Searching Approach

Systematic reviews of methods-based studies may be considered more challenging than reviews of clinical or other non–methods-based studies for several reasons. These include the presence of inconsistent terminology (e.g., different terminology used to describe “external evidence”) and a lack of validated search filters. In addition, methods reviews do not easily fit within a standard PICO (population, intervention, comparators, outcomes) format, which can further complicate designs of traditional search strategies. Therefore, for this review, a comprehensive pearl-growing (CPG) searching approach was deemed suitable (also known as “snowballing” or “citation mining”).^9^ “Pearl growing” refers to a search strategy in which a number of known, relevant studies (i.e., “pearls”) can be used to construct a search strategy to then identify other studies. Then, based on newly identified studies, subsequent searches can be run with additional search terms. The CPG approach leverages a selection of pearls from which suitable databases and search terms can be determined. The review by Jackson et al.^8^ provides a collection of 11 pearls from which an initial search strategy was developed.

Based on the electronic databases in which the pearls were indexed, MEDLINE, MEDLINE In-Process, EMBASE, Web of Science, and EconLit were searched. The first iteration of the search strategy was designed with assistance from both an experienced information specialist and systematic reviewer, with search terms centered around the key themes of “survival,” “sources,” “extrapolation,” and method-specific terminology (e.g., “background mortality”), based on the key terms used in the initially identified pearl papers. Given the broad range of different terminologies, it was anticipated that a relatively large proportion of hits would be excluded (i.e., search sensitivity was favored over specificity). After the first iteration, the search terms were updated to capture any missing terms identified in new “pearls,” and the search was rerun as a second iteration. Both search strategies are provided within the Supplementary Material. Forward and backward citation tracking was also undertaken to complement the formal database searches, to identify any relevant studies that were cited in each of the identified studies as well as subsequent studies that referenced the study after it was published.

In addition to the formal database searching, materials presented at previous meetings of the Professional Society for Health Economics and Outcomes Research (ISPOR) were also considered, through keyword searching via its Presentations Database. Emergent research in the context of HTA methodology has previously been presented at ISPOR meetings, including one of the pearls identified in the Jackson et al.^8^ review (by Guyot et al.^10^).

### Inclusion/Exclusion Criteria and Restrictions

The main inclusion criterion for the review was that studies were required to be “primary” methodological studies, including reference to use of external evidence for the estimation of survival-based outcome(s). In the context of this review, “primary” refers to any study that presents a novel method concerning the use of external evidence in survival extrapolation. Some studies may present an application of, and/or minor alterations to, preexisting methods, neither of which were deemed primary methodological studies for this review. Reviews of methodological studies were included at interim stages because they may contain a novel method developed by the review authors as a result of the review and because such reviews may aid with the identification of other primary studies. However, reviews of methods were ultimately excluded within the presentation of findings, unless as part of the review a primary methodological study was included.

In addition to being a primary methodological study, included studies were also required to make use of external evidence. Here, the term “evidence” is used in preference to “data” or “information” in order to distinguish between different types of evidence that could be used. “Evidence” is used throughout this review to describe both data and information sources that may be used, noting that individual studies may use the terms “data” or “information” when describing specific methods.

Economic evaluations were excluded from consideration, as it was anticipated that the title and/or abstract for the vast majority of published economic evaluations would be unlikely to contain sufficient detail to determine whether or not external evidence had been used to inform the estimation of survival. We deemed it impractical to review records from every economic evaluation and therefore considered economic evaluations to be outside the scope of this review. By limiting our review to methodological studies, we anticipated that we would identify the majority of methods that have been developed to include external evidence within economic evaluations. Methods were also excluded if they did not incorporate external evidence within the estimation of the survival model itself but instead referred to external evidence for validation or contextual purposes.

### Data Extraction and Synthesis

A data extraction template was developed to extract relevant information from the identified studies. Two stages of screening were applied with 2 independent reviews (A.B. and G.S.), with disagreements settled by discussion and with an additional reviewer (N.L.) if needed. Any further disagreements were settled by consensus view via discussion with the remaining members of the review team (M.S. and G.B.). No formal quality assessment of the identified studies was planned, since the purpose of the review was to identify all relevant published methods, regardless of the quality of the studies themselves.

The data extraction template focused on 5 categories: summary information, method requirements, application, validation, and any further commentary. Summary information was extracted to understand key features of the methods (name of the approach, a brief explanation of how the method works, and if the method builds on a previous approach and/or could be grouped with another method). Method requirements were identified to acknowledge what evidence would be required to apply a given method, what key assumptions are made as part of the model-fitting process, and what software package(s) are required. Information regarding the application of the method to a case study was extracted to understand in which contexts the methods had been applied. Information reported on the validation of the methods was recorded to ascertain if the authors had attempted any form of validation of the approach in other settings or compared with other approaches. Finally, further commentary was extracted to document, in the authors’ words, the advantages and disadvantages of their approach.

The protocol for this review was registered as a record with the PROSPERO international prospective register of systematic reviews (CRD42020202034).

## Results

### Study Identification

**Figure 1:**
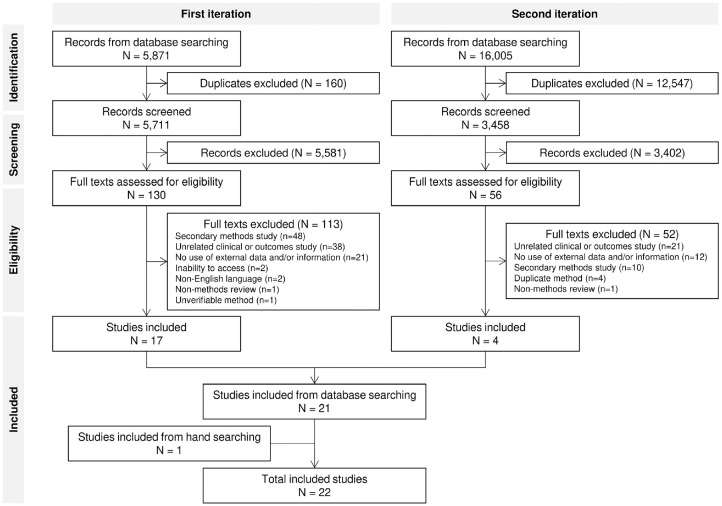
PRISMA flow diagram.

Figure 1 presents a Preferred Reporting Items for Systematic Reviews and Meta-Analyses (PRISMA) diagram for the review. The first search was run on 1 October 2020, with the second search run on 1 July 2021.

For the first iteration, a total of 5,871 records were identified prior to de-duplication. After de-duplication and primary screening based on titles and abstracts, 130 records remained as eligible for secondary screening. As noted previously, a large number of records were expected to be excluded at primary screening owing to the search strategy needing to capture a broad range of terminology. After full-text screening, 113 records were excluded, which left 17 records suitable for data extraction. The second iteration (ran with an updated search strategy based on the findings from the first iteration) resulted in a further 4 records for data extraction. One additional record was identified from hand searching, giving a total of 22 studies for extraction.^6,7,11–30^ From the 22 studies, 18 distinct methods were identified.

### Summary Information for Identified Studies

For the purpose of this review, we sought to categorize methods by common themes that indicated broadly how the external evidence was leveraged within the model-estimation process. However, the categories used are not mutually exclusive, as some methods incorporated multiple elements of approaches to integrate external evidence.

Based on the information extracted from each of the identified method studies, 3 main themes were identified:

- Piecewise methods: These methods involved the use of external evidence to inform extrapolations only after a specified point in time.^6,7,11–15,17,18^
- Methods based on the use of informative prior(s): Several methods included specification of informative priors within a Bayesian framework as a means of using external evidence.^7,15,16,19,20^
- General population adjustment methods: These methods incorporated information relating to general population survival as the source of external evidence.^7,19–27^

In addition to these, a range of “other” methods was also identified, which included some other mechanism related to the use of external evidence and did not fit within a theme.^6,7,17,18,29,30^

**Figure 2:**
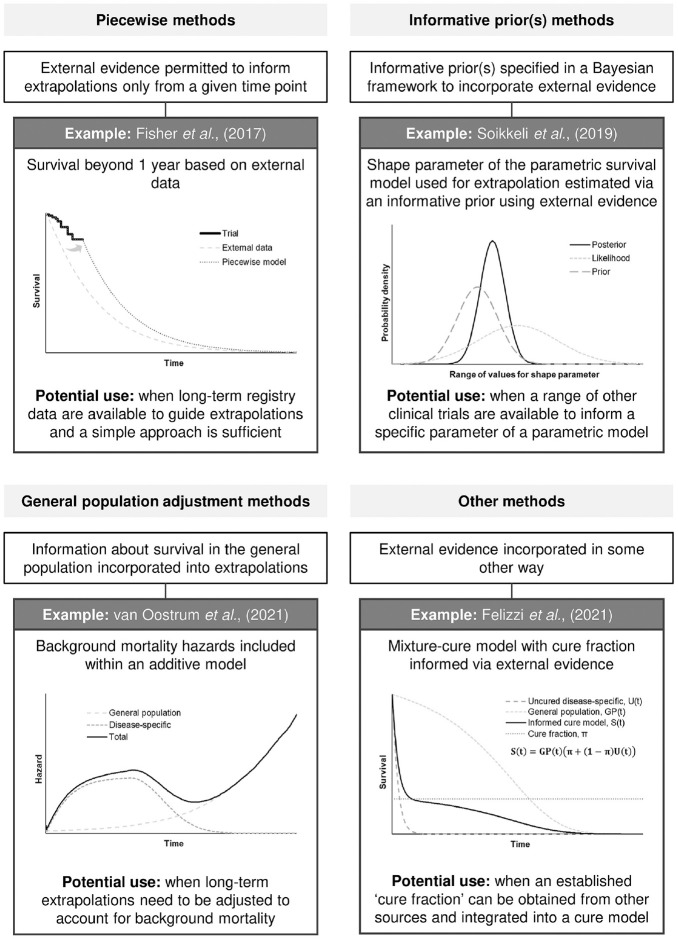
Example of methods for each theme.

Figure 2 provides an example of a method for each of the aforementioned themes (including one example of a method that did not fit within these themes), and in the Supplementary Material, a Sankey diagram illustrates similarities between the methods. The next subsections further describe the features of the identified methods by theme, highlighting individual methods of particular interest. Full details for all identified methods, including data requirements, assumptions, software availability, and whether a frequentist or Bayesian perspective is taken, are provided in the data extraction table (see Supplementary Material). A final subsection is presented that summarizes some key findings across all of the methods identified.

### Piecewise Methods

By restricting the influence of external evidence to affect only estimates beyond a specified cut point, the use of piecewise methods means the external evidence addresses uncertainty in longer-term estimation of survival. The piecewise aspect of these methods may be considered the simplest of the approaches identified to incorporate external evidence, owing to the transparency of how the external evidence is used and because some of these methods can be implemented without specialist software. Nevertheless, their simplicity may also be viewed as their most important limitation, as each piecewise approach makes several strong (and often clinically implausible) assumptions about the pattern of survival in the relevant population (e.g., an abrupt change in hazards at a specific time point).

Several examples of piecewise models were identified, in which the hazard of death was assumed to follow that of an external population from a given time point.^11–13^ However, Nelson et al.^14^ considered a different type of piecewise model that adopted age as a time-based metric for longer-term survival estimation, as opposed to time since study entry. This was the only study identified that used age instead of time as the basis from which to incorporate external evidence and has intuitive appeal to ensure lifetime extrapolations exhibit face validity. Pennington et al.^6^ developed a piecewise model that was capable of using only summary-level data concerning a future point estimate of survival at a specific timepoint (or “landmark”), which could be useful where only limited information is available from an external evidence source.

### Methods Based on the Use of Informative Prior(s)

Methods that involve specification of informative prior(s) allow expectations around survival based upon external evidence to be built into the survival model used for extrapolation. The impact of this approach on extrapolations depends both on the strength of the prior specified and the nature of the evidence being used to inform the prior (and ultimately how this affects survival estimation). For example, constructing a model in which the prior distribution for a given parameter implies that its value is approximately 1, on average, may not influence a model greatly if the parameter was already estimated to be close to 1, whereas a prior that stipulates approximately 10% of patients are alive at 10 y might have a large impact depending on the estimated proportion without a prior.

The most comprehensive of the methods identified under this theme is the multiparameter evidence synthesis approach by Guyot et al.,^7^ which makes use of several different external evidence sources. The other approaches identified allowed informative priors to influence survival extrapolations in a more limited way. For example, Che et al.^15^ specified informative priors in terms of when the external evidence begins to influence the survival extrapolation and how long it takes before survival is explained entirely by the external evidence. Soikkeli et al.^16^ specified a model in which informative priors were imposed on the shape parameter of a parametric survival model fitted to the primary data source, based on the shape parameter value estimated when fitting the same model to different (but related) external data.

These methods can be considered only in a Bayesian framework and so require the use of specialist Bayesian analysis software (e.g., WinBUGS^31^) to be executed efficiently. This may explain, at least in part, their seemingly limited uptake within the HTA field, along with the fact that these methods may be seen as more complicated than frequentist approaches. A further complication is that of transparency regarding how to execute the methods in broader contexts, as these methods require coding expertise to produce extrapolations. As an example, the study by Demiris and Sharples^20^ includes a link to find executable code, but it is no longer valid (which is unsurprising given that Web addresses are likely to change over the course of 15+ y). The need for coding expertise may further dissuade uptake of these methods, despite their advantages.

### General Population Adjustment Methods

Within the general population adjustment methods identified, life table estimates (i.e., national statistics concerning life expectancy in the general population) are assumed to be certain (such that no uncertainty in life table estimates is factored into model fitting), which separates this source of external evidence from other sources. A relative survival approach is featured in several studies, in which disease-specific survival is estimated relative to a background mortality survival estimate. This approach has been applied within the context of a range of parametric modeling approaches, including standard parametric models, restricted cubic spline models, and mixture-cure models, fitted in both a frequentist and Bayesian framework.^20–27^

Outside of a formal relative survival approach, a number of methods were identified in which general population hazards were considered additive, were assumed to eventually converge to those based on a parametric model fitted to the trial data alone, gradually influenced survival estimation according to a “rolling extrapolation” algorithm, or could be used as the basis from which to apply a hazard ratio (HR) to estimate lifetime survival for the population of interest.^27,28^ Each of these approaches considers a different relationship between the hazards for the population under consideration and those of the general population, although the authors of this study suggest that an “internal additive hazards” approach (i.e., a relative survival approach) exhibited the greatest face validity for all data sets they considered.^28^

General population adjustment methods have a distinct advantage compared with methods that rely on other sources of external evidence in that they can be applied for any disease area (and that life tables are readily available for most countries). However, therein lies the fundamental limitation of these methods: adjustments can influence extrapolations based on comparability only to the general population and not the population of interest (i.e., the population with the disease being investigated). Some of these approaches would therefore be expected to have a limited impact on extrapolations, particularly if applied within the context of populations that experience disease-specific mortality far in excess of other-cause mortality.

### Other Methods

The “other” methods were those that used external evidence in a way that did not fit into any of the previously described themes. Examples include the following:

- external evidence used to inform the estimation of a cure fraction within a mixture-cure model,^29^
- models constrained to predict prespecified survival proportions at landmark time points, based on external information,^18^
- parametric models fitted to the primary data source but values “plugged in” for parameters of these models from models fitted to external evidence,^6,30^ and
- methods that based extrapolations on external information that informs expectations of how relative hazard rates (i.e., treatment effects) are likely to change over time.^7,17^

While these methods reflect a broad range of different types of approaches to use external evidence, each imposes specific assumptions and/or requires an explicit decision to be made for how the external evidence can affect survival extrapolation.

Most of these methods were implemented in a frequentist framework, and so the external evidence aspect of the model was treated as “fixed.” In fact, Hawe et al.^30^ presented an approach that may be considered an alternative application of the method presented by Soikkeli et al.,^16^ except within a frequentist approach and making a stronger assumption that parameters taken from one model can be deemed “fixed” and then be applied to estimate other results based on the external evidence. Another example of assuming “fixed” external data was the informed mixture-cure model by Felizzi et al.,^29^ which essentially allowed for the “cure fraction” (that is, a proportion of patients for whom disease-specific mortality is assumed to be zero) to be fixed at a particular value, based on external evidence.

The only method identified in this category that was fitted in a Bayesian framework was the multiparameter evidence synthesis approach by Guyot et al.^7^ (which also features in all of the earlier categories owing to its multifaceted use of external evidence). This method included specifying informative priors on an estimated HR from 6 y, but the value of 6 y was considered a “fixed” input to the model. In this sense, while Bayesian approaches may be criticized for introducing subjective prior information into model fitting, a similar criticism could be made of many of these assumption-based approaches.

## Additional Findings

Most of the methods made use of external data from either another clinical trial, a type of observational study or registry, and/or life tables. Observational studies may provide useful data in a population similar to that of the trial of primary interest yet may suffer from similar limitations to the trial itself, such as small sample sizes and limited durations of follow-up. Registry databases may provide data for a larger number of patients with longer follow-up, but usually these data will reflect a broader patient population. These databases may also include only limited information on factors such as stage of disease, prior treatments, and specific mutations that may be important to consider for HTA, raising issues around the comparability of populations within different data sources.

The methods typically assumed complete generalizability of the population of interest and the external population(s), although some adjusted external data to account for potential imbalances. Within the studies identified, examples were presented mostly reflecting populations with cancer, but some studies included populations with cardiovascular disease (which tended to include larger sample sizes).

No study included an evaluation of a proposed method against another published approach that also made use of external evidence as a means of testing the proposed method, yet applications were presented in several studies to compare against “standard” parametric modeling methods.

## Discussion

External evidence is increasingly used for survival extrapolation within contemporary HTA, particularly given that the National Institute for Health and Care Excellence (NICE) recently launched its Real-World Evidence Framework to aid in its ambition to “use real-world data to resolve gaps in knowledge and drive forward access to innovations for patients.”^32^ This review identified a broad range of different approaches, covering piecewise, informative prior(s), general population adjustment, and “other” approaches. An important finding from our review was that none of the identified studies attempted to evaluate their method against another published approach that also made use of external evidence in a different way. This highlights a broad concern with the use of nontrial evidence in survival extrapolation, that there are a substantial number of approaches that have been put forward in the literature but little in the way of guidance in selecting an appropriate method under a given set of circumstances.

The methods identified as part of this review could theoretically be categorized based on the approach taken to statistical analysis: Bayesian versus frequentist. However, this was not pursued, as it was noted that many of the approaches identified could feasibly be applied within either framework, with the exception of the informative prior(s) methods, which can be implemented only within a Bayesian framework. However, the data extraction table provides details on whether a Bayesian or frequentist perspective was taken (see Supplementary Material). While frequentist approaches may be considered simpler to implement and require less specialized statistical analysis software, a benefit of a Bayesian approach is that it allows for the analyst to control the degree of influence the external evidence is permitted to have on model parameters. Furthermore, a Bayesian analysis more naturally allows the propagation of parameter and model uncertainty through the entire decision model. These are important considerations for HTA, as decision makers should ideally be able to understand the extent to which external evidence affects extrapolations as well as the uncertainty inherent within the analysis.

Owing to the specification of a broad search strategy, a large number of studies were excluded. Some of the excluded studies detailed methods that were adjacent to the topic of survival extrapolation but did not meet the review inclusion criteria. For example, a study by Lewis et al.^33^ highlighted the use of a Bayesian hierarchical survival modeling approach to “borrow” information from historical control data, although the focus of this study was on the estimation of relative effects but without extrapolation of OS and so was excluded. While excluded, elements of these methods feature in the included methods (e.g., “borrowing” from historical control data was a feature of several included methods).^7,11–13,30^

The scope of this review was limited to survival extrapolation methods, but there was a notable overlap in methods used for survival extrapolation and methods used to construct indirect treatment comparisons. For example, Fisher et al.^13,34^ “matched” data from a registry to pivotal trial data to align inclusion criteria, yet matching methods are more commonly used in HTA to reduce the risk of bias when comparing different study populations. As another example, Guyot et al.^10^ made use of HRs to capture an expected duration of treatment effect (one aspect of the multiparameter evidence synthesis), yet HRs are more often the target of inference within a (network) meta-analysis. Estimation of relative treatment effects and extrapolation of survival are intrinsically linked, but methods were included in this review only if they were capable of yielding extrapolations of OS (and not simply measures of relative effects).

Several limitations are acknowledged within the context of this systematic review. First, novel methods that are used only within the context of a de novo economic evaluation were excluded, given that (as previously mentioned) in order to comprehensively identify such methods, one would effectively need to screen all published economic evaluations to determine whether or not external evidence was used. It was therefore not practical to include economic evaluations within this review, which means that it is possible that relevant methods may have been missed. Reassuringly, Jackson et al.^8^ cited only 3 economic evaluations that were known by the authors to contain an external data methods component^35–37^ and contained methods that were already identified from other studies. Therefore, while the exclusion of these types of study is a limitation, it is unlikely that important methods have been missed. However, it would be valuable to investigate which methods are being used in contemporary cost-effectiveness analyses; for this, it is necessary to undertake a different type of review focusing on economic evaluations and then determine from a full-text review which appear to use external evidence and which do not (and for those that do, which methods are used). A separate, standalone review of the contemporary use of external evidence to inform NICE appraisals of cancer drugs has also been undertaken by the study authors.^38^

There is no standard terminology to describe external evidence, and authors used different terminology based on the specific sources cited. Some sources (e.g., a disease-specific registry) may be referenced by name instead of “registry” and so may not have been identified by the search strategy if insufficient detail was otherwise provided in the title or abstract. However, it is likely that disease-specific registries would be referred to in methods papers as a case study and thus described accordingly as a registry within the abstract. In addition, we only considered conference presentations from the ISPOR database, selected as 2 studies were originally presented at an ISPOR meeting before journal publication,^7,17^ and full presentations can be accessed online. There may be methods presented at other meetings that were not identified by our review, but we suspected most conference proceedings would lack the detail required to fully describe a method and that novel methods presented at a conference would likely result in a subsequent paper, and therefore, we do not expect to have missed important methods.

The performance of specific methods was not explicitly evaluated as part of this review, as it is expected that the performance of some methods may be affected greatly by the context in which it has been applied. For example, an age-based piecewise extrapolation method may perform better in an older versus a younger population, because background mortality is likely to have greater influence on survival extrapolations in an older group for whom life expectancy is shorter. Nevertheless, the strengths and weaknesses of each approach (as stated by the study authors) were extracted and summarized in a narrative synthesis, and the extent of evaluation performed by the study authors was assessed (which, at least within the context of comparing to other methods that also use external evidence, was identified as an important omission in the identified studies). Previous studies have assessed a range of extrapolation approaches in cancer populations, although none have included all of the methods described in this review.^17,39–41^ As with the use of methods in the context of economic evaluations, another area for planned future research is a formal evaluation of methods under both real-life circumstances (e.g., through case studies, including economic evaluations) and controlled circumstances (i.e., via a simulation study), including how each method can reflect the inherent uncertainty associated with extrapolations.

## Conclusions

This review reports a summary of possible approaches to incorporate external evidence into survival extrapolations, on the basis of a systematic search of the literature. Several methods were identified with common themes that emerged, namely, methods that adopt a piecewise approach to introduce external evidence beyond a given time point, methods that incorporate external evidence through the specification of informative priors in a Bayesian framework, and methods that introduce general population data to adjust extrapolations. In addition, a number of “other” methods were also identified that rely on some other mechanism to integrate external evidence into model fitting.

An important finding from this review was that no evidence was found comparing the identified methods to one another. This poses a challenge for all parties involved in HTA decision making, ranging from analysts needing to choose and defend a particular approach, to decision makers needing to base their decisions on reliable evidence using appropriate statistical methodology. As such, an assessment of different methods to incorporate external evidence would be a useful area for further research.

On the basis of this review, we recommend that when researchers wish to leverage external evidence for survival extrapolation, they should carefully justify the approach taken to incorporate the evidence and why alternative approaches were not used. This should include details on how the external evidence was identified and the rationale for why the selected source of evidence was chosen (especially if several sources are available). The method used and the analyses applied should also be clearly described, so that reviewers, decision makers, and other stakeholders can understand what external evidence has been used, how it influences estimations, and by how much. At present, it is not possible to make recommendations on the preferred methods for including external evidence to inform extrapolations, but the ultimate objective is to utilize methods that use appropriate evidence in a justifiable way to produce credible extrapolations.

## Supplemental Material

sj-docx-1-mdm-10.1177_0272989X231168618 – Supplemental material for A Systematic Review of Methods to Incorporate External Evidence into Trial-Based Survival Extrapolations for Health Technology Assessment Click here for additional data file. Supplemental material, sj-docx-1-mdm-10.1177_0272989X231168618 for A Systematic Review of Methods to Incorporate External Evidence into Trial-Based Survival Extrapolations for Health Technology Assessment by Ash Bullement, Matthew D. Stevenson, Gianluca Baio, Gemma E. Shields and Nicholas R. Latimer in Medical Decision Making
