# Supplementary material for: A Systematic Review of Methods to Incorporate External Evidence into Trial-Based Survival Extrapolations for Health Technology Assessment
Source: Med Decis Making. 2023 Apr 26;43(5):610–20. doi: 10.1177/0272989X231168618 (PMC10336710; doi:10.1177/0272989X231168618)
Supplement: sj-docx-1-mdm-10.1177_0272989X231168618 – Supplemental material for A Systematic Review of Methods to Incorporate External Evidence into Trial-Based Survival Extrapolations for Health Technology Assessment [file sj-docx-1-mdm-10.1177_0272989X231168618.docx]

# Supplementary material

[Supplementary material 1](#_Toc122683182)

[Search strategies 1](#_Toc122683183)

[Summary Sankey diagram 7](#_Toc122683184)

[Data extraction 9](#_Toc122683185)

## Search strategies

The search strategies employed for this review are provided in the tables that follow. Strategies are presented separately for the first and second searching iterations. Please note that a large number of duplicates were anticipated for the second iteration owing to a combination of (a) studies having been already identified in the first iteration, and (b) the running of the second iteration through Embase and Medline separately.

Table 1: Ovid searching (first iteration)

| # | Search term(s) |
| --- | --- |
| 1 | ((extrap* or estimat* or project* or predict* or expect* or express*) adj3 survival).tw. |
| 2 | (parametric adj2 survival).tw. |
| 3 | (extrap* adj2 (trial* or mortality)).tw. |
| 4 | ((estim* or express*) adj2 life-expectancy).tw. |
| 5 | (RWE or RWD).tw. |
| 6 | ((real-life or real-world) adj3 (data or evidence)).tw. |
| 7 | (external adj (information or data)).tw. |
| 8 | background mortality.tw. |
| 9 | competing risk*.tw. |
| 10 | (excess adj (mortality or hazard*)).tw. |
| 11 | life-table*.tw. |
| 12 | (expert* adj (information or opinion)).tw. |
| 13 | (observational adj2 data*).tw. |
| 14 | ((disease or cause) adj (related or specific) adj (survival* or hazard*)).tw |
| 15 | (registry* or registries).tw. |
| 16 | (reference adj2 population).tw. |
| 17 | or/1-4 |
| 18 | or/5-16 |
| 19 | 17 and 18 |
| 20 | letter.pt. |
| 21 | historical article.pt. |
| 22 | editorial.pt. |
| 23 | conference abstract.pt. |
| 24 | or/20-24 |
| 25 | 19 not 24 |
| 26 | exp animals/ not humans.sh. |
| 27 | 25 not 26 |
| 28 | limit 27 to english language |

Table 2: Web of Science searching (first iteration)

| # | Search term(s) |
| --- | --- |
| 1* | (TI= ((extrap* OR estimat* OR project* OR predict* OR expect* OR express*) near/3 survival) OR AB= ((extrap* OR estimat* OR project* OR predict* OR expect* OR express*) near/3 survival)) |
| 2* | (TI = (parametric near/2 survival) OR AB= (parametric near/2 survival)) |
| 3* | (TI = (extrap* near/2 (trial* or mortality)) OR AB= (extrap* near/2 (trial* or mortality))) |
| 4* | (TI = ((estim* or express*) near/3 (life-expectancy)) OR AB = ((estim* or express*) near/3 (life-expectancy))) |
| 5* | (TI = (RWE or RWD) OR AB= (RWE or RWD)) |
| 6* | (TI = ((real-life or real-world) near/3 (data or evidence)) OR AB= ((real-life or real-world) near/3 (data or evidence))) |
| 7* | ((TI = (external near/1 (information or data))) OR (AB = (external near/1 (information or data)))) |
| 8* | (TI = background-mortality OR AB = background-mortality) |
| 9* | (TI = competing risk* OR AB = competing risk*) |
| 10* | (TI = (excess near/1 (mortality or hazard*))) OR (AB = (excess near/1 (mortality or hazard*))) |
| 11* | (TI = life-table*) OR (AB = life-table*) |
| 12* | (TI = (expert* near/1 (information or opinion))) OR (AB = (expert* near/1 (information or opinion))) |
| 13* | ((TI = (observational near/2 data*)) OR (AB = (observational near/2 data*))) |
| 14* | (TI = ((disease or cause) near/1 (related or specific) near/1 (survival* or hazard*))) OR (AB= ((disease or cause) near/1 (related or specific) near/1 (survival* or hazard*)))) |
| 15* | (TI = (registry* OR registries)) OR (AB = (registry* OR registries)) |
| 16* | (TI = (reference near/2 population)) OR (AB = (reference near/2 population)) |
| 17* | #4 OR #3 OR #2 OR #1 |
| 18* | #16 OR #15 OR #14 OR #13 OR #12 OR #11 OR #10 OR #9 OR #8 OR #7 OR #6 OR #5 |
| 19 | #18 AND #17 **Refined by:** [excluding] databases: (MEDLINE) AND databases: (WOS) *Databases= WOS, BCI, BIOSIS, CCC, DRCI, DIIDW, KJD, MEDLINE, RSCI, SCIELO, ZOOREC Timespan=All years Search language=English* |
| 20^†^ | ANIMAL STUDIES FILTER |
| 21 | (#19 not #20) **Refined by:** document types: (ARTICLE OR EARLY ACCESS OR BOOK OR REVIEW) *Databases= WOS, BCI, BIOSIS, CCC, DRCI, DIIDW, KJD, MEDLINE, RSCI, SCIELO, ZOOREC Timespan=All years Search language=English* |

**Note:** *Each of these lines also include the following additional information: ‘Databases= WOS, BCI, BIOSIS, CCC, DRCI, DIIDW, KJD, MEDLINE, RSCI, SCIELO, ZOOREC Timespan=All years Search language=English’. ^†^Owing to its length, the filter applied to remove animal studies from the search is presented separately later in this appendix for simplicity.

A search filter was used within the first iteration of searching via the Web of Science database to remove animal studies. The filter is based on a previously published PubMed filter by Hooijmans *et al.*, (2010).^1^ The published filter was modified to focus on the specific animal terms that may feature within either the title (‘TI’) or abstract (‘AB’) of animal studies, and aligned with the syntax required to search Web of Science. The filter used is shown below:

(AB = (animal OR animals OR pisces OR fish OR fishes OR catfish OR catfishes OR sheatfish OR silurus OR arius OR heteropneustes OR clarias OR gariepinus OR fathead minnow OR fathead minnows OR pimephales OR promelas OR cichlidae OR trout OR trouts OR char OR chars OR salvelinus OR salmo OR oncorhynchus OR guppy OR guppies OR millionfish OR poecilia OR goldfish OR goldfishes OR carassius OR auratus OR mullet OR mullets OR mugil OR curema OR shark OR sharks OR cod OR cods OR gadus OR morhua OR carp OR carps OR cyprinus OR carpio OR killifish OR eel OR eels OR anguilla OR zander OR sander OR lucioperca OR stizostedion OR turbot OR turbots OR psetta OR flatfish OR flatfishes OR plaice OR pleuronectes OR platessa OR tilapia OR tilapias OR oreochromis OR sarotherodon OR common sole OR dover sole OR solea OR zebrafish OR zebrafishes OR danio OR rerio OR seabass OR dicentrarchus OR labrax OR morone OR lamprey OR lampreys OR petromyzon OR pumpkinseed OR pumpkinseeds OR lepomis OR gibbosus OR herring OR clupea OR harengus OR amphibia OR amphibian OR amphibians OR anura OR salientia OR frog OR frogs OR rana OR toad OR toads OR bufo OR xenopus OR laevis OR bombina OR epidalea OR calamita OR salamander OR salamanders OR newt OR newts OR triturus OR reptilia OR reptile OR reptiles OR bearded dragon OR pogona OR vitticeps OR iguana OR iguanas OR lizard OR lizards OR anguis fragilis OR turtle OR turtles OR snakes OR snake OR aves OR bird OR birds OR quail OR quails OR coturnix OR bobwhite OR colinus OR virginianus OR poultry OR poultries OR fowl OR fowls OR chicken OR chickens OR gallus OR zebra finch OR taeniopygia OR guttata OR canary OR canaries OR serinus OR canaria OR parakeet OR parakeets OR grasskeet OR parrot OR parrots OR psittacine OR psittacines OR shelduck OR tadorna OR goose OR geese OR branta OR leucopsis OR woodlark OR lullula OR flycatcher OR ficedula OR hypoleuca OR dove OR doves OR geopelia OR cuneata OR duck OR ducks OR greylag OR graylag OR anser OR harrier OR circus pygargus OR red knot OR great knot OR calidris OR canutus OR godwit OR limosa OR lapponica OR meleagris OR gallopavo OR jackdaw OR corvus OR monedula OR ruff OR philomachus OR pugnax OR lapwing OR peewit OR plover OR vanellus OR swan OR cygnus OR columbianus OR bewickii OR gull OR chroicocephalus OR ridibundus OR albifrons OR great tit OR parus OR aythya OR fuligula OR streptopelia OR risoria OR spoonbill OR platalea OR leucorodia OR blackbird OR turdus OR merula OR blue tit OR cyanistes OR pigeon OR pigeons OR columba OR pintail OR anas OR starling OR sturnus OR owl OR athene noctua OR pochard OR ferina OR cockatiel OR nymphicus OR hollandicus OR skylark OR alauda OR tern OR sterna OR teal OR crecca OR oystercatcher OR haematopus OR ostralegus OR shrew OR shrews OR sorex OR araneus OR crocidura OR russula OR european mole OR talpa OR chiroptera OR bat OR bats OR eptesicus OR serotinus OR myotis OR dasycneme OR daubentonii OR pipistrelle OR pipistrellus OR cat OR cats OR felis OR catus OR feline OR dog OR dogs OR canis OR canine OR canines OR otter OR otters OR lutra OR badger OR badgers OR meles OR fitchew OR fitch OR foumart or foulmart OR ferrets OR ferret OR polecat OR polecats OR mustela OR putorius OR weasel OR weasels OR fox OR foxes OR vulpes OR common seal OR phoca OR vitulina OR grey seal OR halichoerus OR horse OR horses OR equus OR equine OR equidae OR donkey OR donkeys OR mule OR mules OR pig OR pigs OR swine OR swines OR hog OR hogs OR boar OR boars OR porcine OR piglet OR piglets OR sus OR scrofa OR llama OR llamas OR lama OR glama OR deer OR deers OR cervus OR elaphus OR cow OR cows OR bos taurus OR bos indicus OR bovine OR bull OR bulls OR cattle OR bison OR bisons OR sheep OR sheeps OR ovis aries OR ovine OR lamb OR lambs OR mouflon OR mouflons OR goat OR goats OR capra OR caprine OR chamois OR rupicapra OR leporidae OR lagomorpha OR lagomorph OR rabbit OR rabbits OR oryctolagus OR cuniculus OR laprine OR hares OR lepus OR rodentia OR rodent OR rodents OR murinae OR mouse OR mice OR mus OR musculus OR murine OR woodmouse OR apodemus OR rat OR rats OR rattus OR norvegicus OR guinea pig OR guinea pigs OR cavia OR porcellus OR hamster OR hamsters OR mesocricetus OR cricetulus OR cricetus OR gerbil OR gerbils OR jird OR jirds OR meriones OR unguiculatus OR jerboa OR jerboas OR jaculus OR chinchilla OR chinchillas OR beaver OR beavers OR castor fiber OR castor canadensis OR sciuridae OR squirrel OR squirrels OR sciurus OR chipmunk OR chipmunks OR marmot OR marmots OR marmota OR suslik OR susliks OR spermophilus OR cynomys OR cottonrat OR cottonrats OR sigmodon OR vole OR voles OR microtus OR myodes OR glareolus OR primate OR primates OR prosimian OR prosimians OR lemur OR lemurs OR lemuridae OR loris OR bush baby OR bush babies OR bushbaby OR bushbabies OR galago OR galagos OR anthropoidea OR anthropoids OR simian OR simians OR monkey OR monkeys OR marmoset OR marmosets OR callithrix OR cebuella OR tamarin OR tamarins OR saguinus OR leontopithecus OR squirrel monkey OR squirrel monkeys OR saimiri OR night monkey OR night monkeys OR owl monkey OR owl monkeys OR douroucoulis OR aotus OR spider monkey OR spider monkeys OR ateles OR baboon OR baboons OR papio OR rhesus monkey OR macaque OR macaca OR mulatta OR cynomolgus OR fascicularis OR green monkey OR green monkeys OR chlorocebus OR vervet OR vervets OR pygerythrus OR hominoidea OR ape OR apes OR hylobatidae OR gibbon OR gibbons OR siamang OR siamangs OR nomascus OR symphalangus OR hominidae OR orangutan OR orangutans OR pongo OR chimpanzee OR chimpanzees OR pan troglodytes OR bonobo OR bonobos OR pan paniscus OR gorilla OR gorillas OR troglodytes) OR TI = (animal OR animals OR pisces OR fish OR fishes OR catfish OR catfishes OR sheatfish OR silurus OR arius OR heteropneustes OR clarias OR gariepinus OR fathead minnow OR fathead minnows OR pimephales OR promelas OR cichlidae OR trout OR trouts OR char OR chars OR salvelinus OR salmo OR oncorhynchus OR guppy OR guppies OR millionfish OR poecilia OR goldfish OR goldfishes OR carassius OR auratus OR mullet OR mullets OR mugil OR curema OR shark OR sharks OR cod OR cods OR gadus OR morhua OR carp OR carps OR cyprinus OR carpio OR killifish OR eel OR eels OR anguilla OR zander OR sander OR lucioperca OR stizostedion OR turbot OR turbots OR psetta OR flatfish OR flatfishes OR plaice OR pleuronectes OR platessa OR tilapia OR tilapias OR oreochromis OR sarotherodon OR common sole OR dover sole OR solea OR zebrafish OR zebrafishes OR danio OR rerio OR seabass OR dicentrarchus OR labrax OR morone OR lamprey OR lampreys OR petromyzon OR pumpkinseed OR pumpkinseeds OR lepomis OR gibbosus OR herring OR clupea OR harengus OR amphibia OR amphibian OR amphibians OR anura OR salientia OR frog OR frogs OR rana OR toad OR toads OR bufo OR xenopus OR laevis OR bombina OR epidalea OR calamita OR salamander OR salamanders OR newt OR newts OR triturus OR reptilia OR reptile OR reptiles OR bearded dragon OR pogona OR vitticeps OR iguana OR iguanas OR lizard OR lizards OR anguis fragilis OR turtle OR turtles OR snakes OR snake OR aves OR bird OR birds OR quail OR quails OR coturnix OR bobwhite OR colinus OR virginianus OR poultry OR poultries OR fowl OR fowls OR chicken OR chickens OR gallus OR zebra finch OR taeniopygia OR guttata OR canary OR canaries OR serinus OR canaria OR parakeet OR parakeets OR grasskeet OR parrot OR parrots OR psittacine OR psittacines OR shelduck OR tadorna OR goose OR geese OR branta OR leucopsis OR woodlark OR lullula OR flycatcher OR ficedula OR hypoleuca OR dove OR doves OR geopelia OR cuneata OR duck OR ducks OR greylag OR graylag OR anser OR harrier OR circus pygargus OR red knot OR great knot OR calidris OR canutus OR godwit OR limosa OR lapponica OR meleagris OR gallopavo OR jackdaw OR corvus OR monedula OR ruff OR philomachus OR pugnax OR lapwing OR peewit OR plover OR vanellus OR swan OR cygnus OR columbianus OR bewickii OR gull OR chroicocephalus OR ridibundus OR albifrons OR great tit OR parus OR aythya OR fuligula OR streptopelia OR risoria OR spoonbill OR platalea OR leucorodia OR blackbird OR turdus OR merula OR blue tit OR cyanistes OR pigeon OR pigeons OR columba OR pintail OR anas OR starling OR sturnus OR owl OR athene noctua OR pochard OR ferina OR cockatiel OR nymphicus OR hollandicus OR skylark OR alauda OR tern OR sterna OR teal OR crecca OR oystercatcher OR haematopus OR ostralegus OR shrew OR shrews OR sorex OR araneus OR crocidura OR russula OR european mole OR talpa OR chiroptera OR bat OR bats OR eptesicus OR serotinus OR myotis OR dasycneme OR daubentonii OR pipistrelle OR pipistrellus OR cat OR cats OR felis OR catus OR feline OR dog OR dogs OR canis OR canine OR canines OR otter OR otters OR lutra OR badger OR badgers OR meles OR fitchew OR fitch OR foumart or foulmart OR ferrets OR ferret OR polecat OR polecats OR mustela OR putorius OR weasel OR weasels OR fox OR foxes OR vulpes OR common seal OR phoca OR vitulina OR grey seal OR halichoerus OR horse OR horses OR equus OR equine OR equidae OR donkey OR donkeys OR mule OR mules OR pig OR pigs OR swine OR swines OR hog OR hogs OR boar OR boars OR porcine OR piglet OR piglets OR sus OR scrofa OR llama OR llamas OR lama OR glama OR deer OR deers OR cervus OR elaphus OR cow OR cows OR bos taurus OR bos indicus OR bovine OR bull OR bulls OR cattle OR bison OR bisons OR sheep OR sheeps OR ovis aries OR ovine OR lamb OR lambs OR mouflon OR mouflons OR goat OR goats OR capra OR caprine OR chamois OR rupicapra OR leporidae OR lagomorpha OR lagomorph OR rabbit OR rabbits OR oryctolagus OR cuniculus OR laprine OR hares OR lepus OR rodentia OR rodent OR rodents OR murinae OR mouse OR mice OR mus OR musculus OR murine OR woodmouse OR apodemus OR rat OR rats OR rattus OR norvegicus OR guinea pig OR guinea pigs OR cavia OR porcellus OR hamster OR hamsters OR mesocricetus OR cricetulus OR cricetus OR gerbil OR gerbils OR jird OR jirds OR meriones OR unguiculatus OR jerboa OR jerboas OR jaculus OR chinchilla OR chinchillas OR beaver OR beavers OR castor fiber OR castor canadensis OR sciuridae OR squirrel OR squirrels OR sciurus OR chipmunk OR chipmunks OR marmot OR marmots OR marmota OR suslik OR susliks OR spermophilus OR cynomys OR cottonrat OR cottonrats OR sigmodon OR vole OR voles OR microtus OR myodes OR glareolus OR primate OR primates OR prosimian OR prosimians OR lemur OR lemurs OR lemuridae OR loris OR bush baby OR bush babies OR bushbaby OR bushbabies OR galago OR galagos OR anthropoidea OR anthropoids OR simian OR simians OR monkey OR monkeys OR marmoset OR marmosets OR callithrix OR cebuella OR tamarin OR tamarins OR saguinus OR leontopithecus OR squirrel monkey OR squirrel monkeys OR saimiri OR night monkey OR night monkeys OR owl monkey OR owl monkeys OR douroucoulis OR aotus OR spider monkey OR spider monkeys OR ateles OR baboon OR baboons OR papio OR rhesus monkey OR macaque OR macaca OR mulatta OR cynomolgus OR fascicularis OR green monkey OR green monkeys OR chlorocebus OR vervet OR vervets OR pygerythrus OR hominoidea OR ape OR apes OR hylobatidae OR gibbon OR gibbons OR siamang OR siamangs OR nomascus OR symphalangus OR hominidae OR orangutan OR orangutans OR pongo OR chimpanzee OR chimpanzees OR pan troglodytes OR bonobo OR bonobos OR pan paniscus OR gorilla OR gorillas OR troglodytes))

Databases= WOS, BCI, BIOSIS, CCC, DRCI, DIIDW, KJD, MEDLINE, RSCI, SCIELO, ZOOREC Timespan=All years Search language=English

Table 3: Ovid searching (second iteration, Embase)

| # | Search term(s) |
| --- | --- |
| 1 | ((extrap* or estimat* or project* or predict* or expect* or express*) adj3 survival).tw. |
| 2^a^ | ((parametric adj2 survival) or (surviv* adj2 (model* or curve*))).tw. |
| 3 | (extrap* adj2 (trial* or mortality)).tw. |
| 4 | ((estim* or express*) adj2 life-expectancy).tw. |
| 5^b^ | ((Bayesian adj (model* or framework or surviv*)) or (prior* adj3 (data or information or hazard*))).tw. |
| 6^c^ | (((real-life or real-world) adj3 (data or evidence)) or (RWE or RWD)).tw. |
| 7 | (external adj (information or data)).tw. |
| 8^c^ | (background mortality or life-table).tw. |
| 9 | competing risk*.tw. |
| 10 | (excess adj (mortality or hazard*)).tw. |
| 11^d^ | (historical and (data or trial or control or evidence)).tw. |
| 12 | (expert* adj (information or opinion)).tw. |
| 13 | (observational adj2 data*).tw. |
| 14^e^ | ((((disease or cause) adj (related or specific)) or baseline) adj (survival* or hazard*)).tw. |
| 15 | (registry* or registries).tw. |
| 16 | (reference adj2 population).tw. |
| 17 | or/1-4 |
| 18 | or/5-16 |
| 19 | 17 and 18 |
| 20^c^ | (letter or historical article or editorial or conference abstract).pt. |
| 21^c^ | 19 not 20 |
| 22 | exp animals/ not humans.sh. |
| 23 | 21 not 22 |
| 24 | limit 23 to english language |
| 25^f^ | 24 not nomogram.tw. |

**Note:** ^a^ Line added in acknowledgement of some studies referring to survival models or survival curves, without saying “parametric”. Some papers described extrapolating survival curves, or even abbreviated this to just extrapolating curves. ^b^ The previous strategy did not include any specific terms related to Bayesian terminology, and therefore was likely to have missed some relevant studies. This line was added to capture studies using these terms. ^c^ Some lines were combined and condensed in the interest of brevity, but the content of the search terms themselves was left unchanged. ^d^ This line was added in to address the fact that the term “historical” was sometimes used to described external information/ data. ^e^ This line was edited to acknowledge some studies referring to “baseline survival” or “baseline hazards”. ^f^ Added to remove nomogram studies that are not relevant. This increased specificity without compromising sensitivity of the search.

Table 4: Ovid searching (second iteration, Medline and EconLit)

| # | Search term(s) |
| --- | --- |
| 1 | ((extrap* or estimat* or project* or predict* or expect* or express*) adj3 survival).tw. |
| 2^a^ | ((parametric adj2 survival) or (surviv* adj2 (model* or curve*))).tw. |
| 3 | (extrap* adj2 (trial* or mortality)).tw. |
| 4 | ((estim* or express*) adj2 life-expectancy).tw. |
| 5^b^ | ((Bayesian adj (model* or framework or surviv*)) or (prior* adj3 (data or information or hazard*))).tw. |
| 6^c^ | (((real-life or real-world) adj3 (data or evidence)) or (RWE or RWD)).tw. |
| 7 | (external adj (information or data)).tw. |
| 8^c^ | (background mortality or life-table).tw. |
| 9 | competing risk*.tw. |
| 10 | (excess adj (mortality or hazard*)).tw. |
| 11^d^ | (historical and (data or trial or control or evidence)).tw. |
| 12 | (expert* adj (information or opinion)).tw. |
| 13 | (observational adj2 data*).tw. |
| 14^e^ | ((((disease or cause) adj (related or specific)) or baseline) adj (survival* or hazard*)).tw. |
| 15 | (registry* or registries).tw. |
| 16 | (reference adj2 population).tw. |
| 17 | or/1-4 |
| 18 | or/5-16 |
| 19 | 17 and 18 |
| 20^c^ | (letter or historical article or editorial or conference abstract).pt. |
| 21^c^ | 19 not 20 |
| 22 | exp animals/ not humans.sh. |
| 23 | 21 not 22 |
| 24 | limit 23 to english language |
| 25^f^ | 24 not nomogram.tw. |

**Note:** ^a^ Line added in acknowledgement of some studies referring to survival models or survival curves, without saying “parametric”. Some papers described extrapolating survival curves, or even abbreviated this to just extrapolating curves. ^b^ The previous strategy did not include any specific terms related to Bayesian terminology, and therefore was likely to have missed some relevant studies. This line was added to capture studies using these terms. ^c^ Some lines were combined and condensed in the interest of brevity, but the content of the search terms themselves was left unchanged. ^d^ This line was added in to address the fact that the term “historical” was sometimes used to described external information/ data. ^e^ This line was edited to acknowledge some studies referring to “baseline survival” or “baseline hazards”. ^f^ Added to remove nomogram studies that are not relevant. This increased specificity without compromising sensitivity of the search.

Table 5: Web of Science searching (second iteration)

| # | Search term(s) |
| --- | --- |
| 1* | TS = (((extrap* OR estimat* OR project* OR predict* OR expect* OR express*) near/3 survival)) |
| 2* | TS = (((parametric near/2 survival) OR (surviv* near/2 (model* OR curve*)))) |
| 3* | TS = (extrap* near/2 (trial* or mortality)) |
| 4* | TS = ((estim* or express*) near/3 (life-expectancy)) |
| 5* | TS = (((Bayesian near/1 (model* or framework or surviv*)) or (prior* near/3 (data or information or hazard*)))) |
| 6* | TS = ((((real-life or real-world) near/3 (data or evidence)) or (RWE or RWD))) |
| 7* | TS = ((external near/1 (information or data))) |
| 8* | TS = (background-mortality or life-table) |
| 9* | TS = (competing risk*) |
| 10* | TS = (excess near/1 (mortality or hazard*)) |
| 11* | TS = (historical and (data or trial or control or evidence)) |
| 12* | TS = (expert* near/1 (information or opinion)) |
| 13* | TS = (observational near/2 data*) |
| 14* | TS = ((((disease or cause) near/1 (related or specific)) or baseline) near/1 (survival* or hazard*)) |
| 15* | TS = (registry* OR registries) |
| 16* | TS = (reference near/2 population) |
| 17* | #4 OR #3 OR #2 OR #1 |
| 18* | #16 OR #15 OR #14 OR #13 OR #12 OR #11 OR #10 OR #9 OR #8 OR #7 OR #6 OR #5 |
| 19 | #18 AND #17 **Refined by:** [excluding] databases: (MEDLINE) AND databases: (WOS) *Databases= WOS, BCI, BIOSIS, CCC, DRCI, DIIDW, KJD, MEDLINE, RSCI, SCIELO, ZOOREC Timespan=All years Search language=English* |
| 20^†^ | ANIMAL STUDIES FILTER |
| 21 | (#19 not #20) **Refined by:** document types: (ARTICLE OR EARLY ACCESS OR BOOK OR REVIEW) *Databases= WOS, BCI, BIOSIS, CCC, DRCI, DIIDW, KJD, MEDLINE, RSCI, SCIELO, ZOOREC Timespan=All years Search language=English* |

**Note:** *Each of these lines also include the following additional information: ‘Databases= WOS, BCI, BIOSIS, CCC, DRCI, DIIDW, KJD, MEDLINE, RSCI, SCIELO, ZOOREC Timespan=All years Search language=English’. ^†^Owing to its length, the filter applied to remove animal studies from the search is presented separately elsewhere in this appendix for simplicity.

For the second iteration, the original filter was simplified to instead search based on topic (‘TS’), which searches the title, abstract, author keywords, and Keywords Plus^®^ (a set of key words unique to the Web of Science database, which includes words and phrases that have been harvested from the titles of cited articles). This edit was made in the interest of efficiency in case the search was to be repeated in the future. The revised animal filter is shown below:

(TS = (animal OR animals OR pisces OR fish OR fishes OR catfish OR catfishes OR sheatfish OR silurus OR arius OR heteropneustes OR clarias OR gariepinus OR fathead minnow OR fathead minnows OR pimephales OR promelas OR cichlidae OR trout OR trouts OR char OR chars OR salvelinus OR salmo OR oncorhynchus OR guppy OR guppies OR millionfish OR poecilia OR goldfish OR goldfishes OR carassius OR auratus OR mullet OR mullets OR mugil OR curema OR shark OR sharks OR cod OR cods OR gadus OR morhua OR carp OR carps OR cyprinus OR carpio OR killifish OR eel OR eels OR anguilla OR zander OR sander OR lucioperca OR stizostedion OR turbot OR turbots OR psetta OR flatfish OR flatfishes OR plaice OR pleuronectes OR platessa OR tilapia OR tilapias OR oreochromis OR sarotherodon OR common sole OR dover sole OR solea OR zebrafish OR zebrafishes OR danio OR rerio OR seabass OR dicentrarchus OR labrax OR morone OR lamprey OR lampreys OR petromyzon OR pumpkinseed OR pumpkinseeds OR lepomis OR gibbosus OR herring OR clupea OR harengus OR amphibia OR amphibian OR amphibians OR anura OR salientia OR frog OR frogs OR rana OR toad OR toads OR bufo OR xenopus OR laevis OR bombina OR epidalea OR calamita OR salamander OR salamanders OR newt OR newts OR triturus OR reptilia OR reptile OR reptiles OR bearded dragon OR pogona OR vitticeps OR iguana OR iguanas OR lizard OR lizards OR anguis fragilis OR turtle OR turtles OR snakes OR snake OR aves OR bird OR birds OR quail OR quails OR coturnix OR bobwhite OR colinus OR virginianus OR poultry OR poultries OR fowl OR fowls OR chicken OR chickens OR gallus OR zebra finch OR taeniopygia OR guttata OR canary OR canaries OR serinus OR canaria OR parakeet OR parakeets OR grasskeet OR parrot OR parrots OR psittacine OR psittacines OR shelduck OR tadorna OR goose OR geese OR branta OR leucopsis OR woodlark OR lullula OR flycatcher OR ficedula OR hypoleuca OR dove OR doves OR geopelia OR cuneata OR duck OR ducks OR greylag OR graylag OR anser OR harrier OR circus pygargus OR red knot OR great knot OR calidris OR canutus OR godwit OR limosa OR lapponica OR meleagris OR gallopavo OR jackdaw OR corvus OR monedula OR ruff OR philomachus OR pugnax OR lapwing OR peewit OR plover OR vanellus OR swan OR cygnus OR columbianus OR bewickii OR gull OR chroicocephalus OR ridibundus OR albifrons OR great tit OR parus OR aythya OR fuligula OR streptopelia OR risoria OR spoonbill OR platalea OR leucorodia OR blackbird OR turdus OR merula OR blue tit OR cyanistes OR pigeon OR pigeons OR columba OR pintail OR anas OR starling OR sturnus OR owl OR athene noctua OR pochard OR ferina OR cockatiel OR nymphicus OR hollandicus OR skylark OR alauda OR tern OR sterna OR teal OR crecca OR oystercatcher OR haematopus OR ostralegus OR shrew OR shrews OR sorex OR araneus OR crocidura OR russula OR european mole OR talpa OR chiroptera OR bat OR bats OR eptesicus OR serotinus OR myotis OR dasycneme OR daubentonii OR pipistrelle OR pipistrellus OR cat OR cats OR felis OR catus OR feline OR dog OR dogs OR canis OR canine OR canines OR otter OR otters OR lutra OR badger OR badgers OR meles OR fitchew OR fitch OR foumart or foulmart OR ferrets OR ferret OR polecat OR polecats OR mustela OR putorius OR weasel OR weasels OR fox OR foxes OR vulpes OR common seal OR phoca OR vitulina OR grey seal OR halichoerus OR horse OR horses OR equus OR equine OR equidae OR donkey OR donkeys OR mule OR mules OR pig OR pigs OR swine OR swines OR hog OR hogs OR boar OR boars OR porcine OR piglet OR piglets OR sus OR scrofa OR llama OR llamas OR lama OR glama OR deer OR deers OR cervus OR elaphus OR cow OR cows OR bos taurus OR bos indicus OR bovine OR bull OR bulls OR cattle OR bison OR bisons OR sheep OR sheeps OR ovis aries OR ovine OR lamb OR lambs OR mouflon OR mouflons OR goat OR goats OR capra OR caprine OR chamois OR rupicapra OR leporidae OR lagomorpha OR lagomorph OR rabbit OR rabbits OR oryctolagus OR cuniculus OR laprine OR hares OR lepus OR rodentia OR rodent OR rodents OR murinae OR mouse OR mice OR mus OR musculus OR murine OR woodmouse OR apodemus OR rat OR rats OR rattus OR norvegicus OR guinea pig OR guinea pigs OR cavia OR porcellus OR hamster OR hamsters OR mesocricetus OR cricetulus OR cricetus OR gerbil OR gerbils OR jird OR jirds OR meriones OR unguiculatus OR jerboa OR jerboas OR jaculus OR chinchilla OR chinchillas OR beaver OR beavers OR castor fiber OR castor canadensis OR sciuridae OR squirrel OR squirrels OR sciurus OR chipmunk OR chipmunks OR marmot OR marmots OR marmota OR suslik OR susliks OR spermophilus OR cynomys OR cottonrat OR cottonrats OR sigmodon OR vole OR voles OR microtus OR myodes OR glareolus OR primate OR primates OR prosimian OR prosimians OR lemur OR lemurs OR lemuridae OR loris OR bush baby OR bush babies OR bushbaby OR bushbabies OR galago OR galagos OR anthropoidea OR anthropoids OR simian OR simians OR monkey OR monkeys OR marmoset OR marmosets OR callithrix OR cebuella OR tamarin OR tamarins OR saguinus OR leontopithecus OR squirrel monkey OR squirrel monkeys OR saimiri OR night monkey OR night monkeys OR owl monkey OR owl monkeys OR douroucoulis OR aotus OR spider monkey OR spider monkeys OR ateles OR baboon OR baboons OR papio OR rhesus monkey OR macaque OR macaca OR mulatta OR cynomolgus OR fascicularis OR green monkey OR green monkeys OR chlorocebus OR vervet OR vervets OR pygerythrus OR hominoidea OR ape OR apes OR hylobatidae OR gibbon OR gibbons OR siamang OR siamangs OR nomascus OR symphalangus OR hominidae OR orangutan OR orangutans OR pongo OR chimpanzee OR chimpanzees OR pan troglodytes OR bonobo OR bonobos OR pan paniscus OR gorilla OR gorillas OR troglodytes))

Databases= WOS, BCI, BIOSIS, CCC, DRCI, DIIDW, KJD, MEDLINE, RSCI, SCIELO, ZOOREC Timespan=All years Search language=English

## Summary Sankey diagram

Figure 1 presents a Sankey diagram of the common themes across the identified methods. For further information concerning each of these methods, please refer to the Data extraction sub-section of the supplementary material. For brevity, names were assigned to each identified method based on the overall description of the approach provided by the authors.

Figure 1: Sankey diagram illustrating categorisation of identified methods


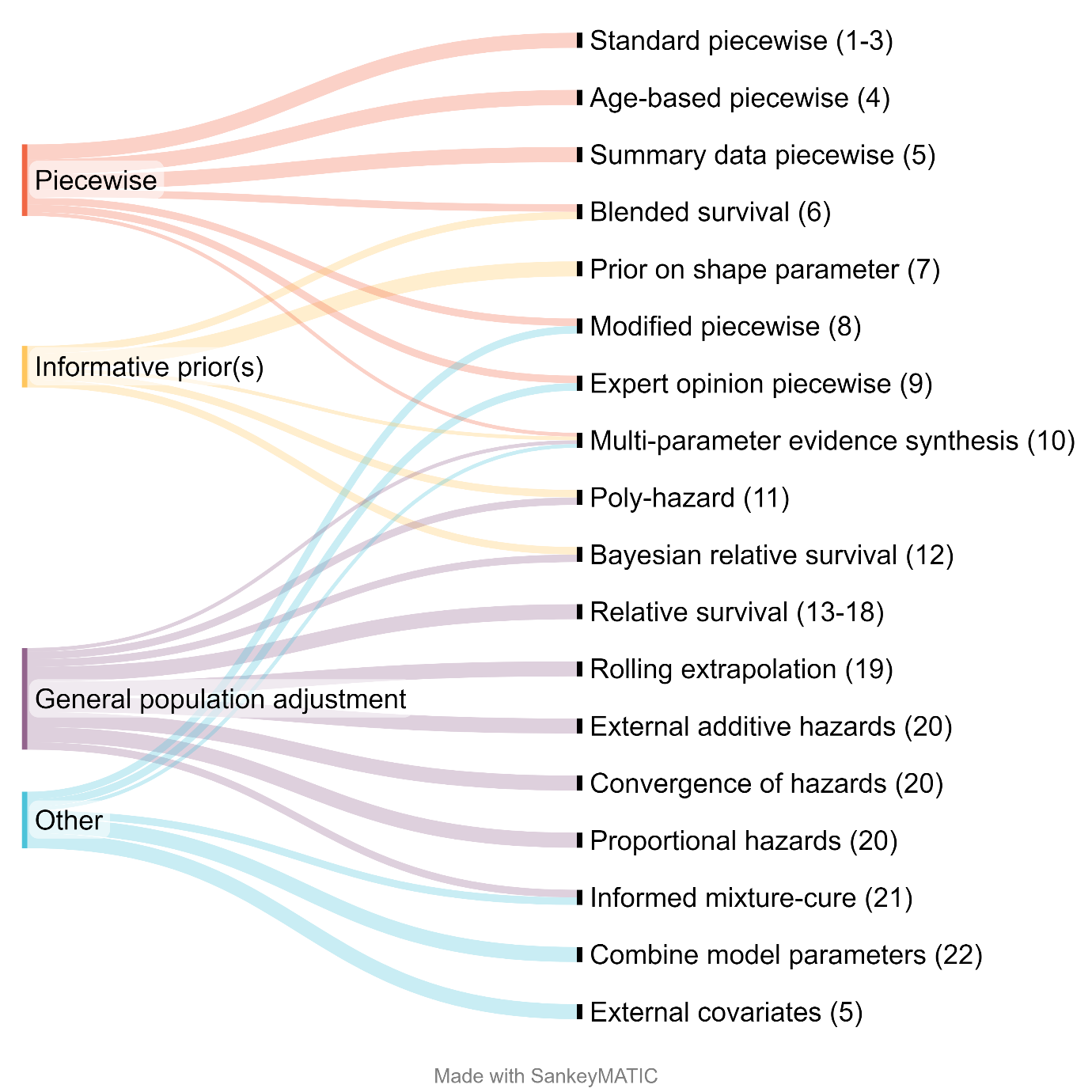


**Note:** Diagram created using SankeyMATIC, available at: <https://sankeymatic.com/build/>.

**Sources:** (1) Larkin *et al.*, 2015^2^; (2) Barlev *et al.*, 2017^3^; (3) Fisher *et al.*, 2017^4^; (4) Nelson *et al.*, 2008^5^; (5) Pennington *et al.*, 2018^6^; (6) Che *et al.*, 2021^7^; (7) Soikkeli *et al.*, 2019^8^; (8) Vickers, 2019^9^; (9) Cope *et al.*, 2019^10^; (10) Guyot *et al.*, 2017^11^; (11) Benaglia *et al.*, 2015^12^; (12) Demiris & Sharples, 2006^13^; (13) Lambert *et al.*, 2005^14^; (14) Lambert *et al.*, 2007^15^; (15) Lambert *et al.*, 2010^16^; (16) Nelson *et al.*, 2007^17^; (17) Andersson *et al.*, 2013^18^; (18) Jakobsen *et al.*, 2019^19^; (19) Hwang *et al.*, 2017^20^; (20) van Oostrum *et al.*, 2021^21^; (21) Felizzi *et al.*, 2021^22^; (22) Hawe *et al.*, 2014^23^.

##

## Data extraction

For ease of presentation, the full data extraction table is presented as two separate tables. The first table, Table 6, presents information about the original article information, key information about the method itself, and some important requirements related to software packages and data inputs. The second table, Table 7, presents further information about how the method was presented in the original article, how it was evaluated, and some additional commentary.

Table 6: Data extraction (Part 1 of 2)

| Article info | | Summary information | | | Requirements | | |
| --- | --- | --- | --- | --- | --- | --- | --- |
| Author(s) | Year | Name(s) | Description of method | Extension of method? | Data required | Key assumptions | Statistical software |
| Larkin *et al.* | 2015 | Standard piecewise | Three-part curve fit was used:  • From 0–1.5 years, Kaplan–Meier estimate from the trial  • From 1.5–5 years, standard parametric curve fits  • After 5 years, long-term data from the AJCC registry | No | PLD for index population + observational data source (digitised KM estimate) | None described by authors, but main assumption is generalisability of source for long-term hazards from registry data  Frequentist method | None listed |
| Barlev *et al.* | 2017 | Standard piecewise | Three stages:  • KM from trial  • Conditional survival from an observational source  • Life tables assuming cure after 5 years | No | PLD for index population + observational data source + life tables | Assumes no benefit in terms of HR after end of KM period + assumes cure after 5 years  Frequentist method | None listed |
| Fisher *et al.* | 2017 | Standard piecewise | Two stages:  • KM from pooled trial data  • Parametric model fitted to registry data | No | PLD for index population + observational data source | Assumes generalisability of conditional survival between trial + observational study sources  Frequentist method | Fitted using Stata and MS Excel |
| Demiris & Sharples | 2006 | Bayesian relative survival (additive hazard and multiplicative hazard models) | Looked at models based on parametric/semi-parametric versions of additive/multiplicative hazard models. Two sources of data: "trial" and "life tables". Priors set for baseline hazard using life table data, and for effect of ICD based on the "trial" data. Meta-analysis output used to obtain an estimate of the HR for ICD versus AAD. PH assumed | Yes, considered to build on literature for relative survival | Two data sources: (1) PLD for index population, and (2) reference data for the general population. Also published HRs to allow curves to be produced for other treatment arms | Method assumes that long term survival distributions for patients have a similar shape to age–sex matched controls from an external source (such as census data), but have a fixed increase in risk, either additive or multiplicative. For relative effect, approach assumes PH by specifying a constant HR  Bayesian method | Authors note that all the methods described in the paper can be fitted using WinBUGS |
| Benaglia *et al.* | 2015 | Poly-hazard | Paper predominantly concerned with the poly-Weibull or poly-Gompertz models (i.e., models that can be fitted within a PH framework). The method involves considering that there are two potential causes of death (e.g., disease-related, or non-disease-related). Method assumes the non-disease-related hazards are identical to an external data set. Then, assuming PH hold between the risks of death (i.e., risk of death in the study group is proportionally higher than in the other data set), can fit a poly-hazard model | Yes, considered to be extensions of standard parametric models (e.g., Weibull) | Two PLD datasets, one from the general population and one for index population. Method assumes that the different causes of death are known in the non-trial data source (and can be quantified) | For an individual in the general population, the distributions of the times to death for two causes come from the same parametric family but with different parameters. Poly-Weibull assumes that hazards for different causes are additive and independent of each other, an assumption that is impossible to test and may require sensitivity analysis  Bayesian method | Fitted in WinBUGS |
| Guyot *et al.* | 2017 | Multi-parameter evidence synthesis | Survival models fitted within a Bayesian framework, with various priors/ restrictions set: • OS at 40 years ≤ General population survival at 40 years • Conditional survival after 6 years assumed identical between trial and SEER data • Conditional survival after 33 years assumed equal to general population (to stop general population and SEER sources conflicting) • HR up to year 5 based on a spline model • HR from year 6 onwards was roughly =1, with some uncertainty built into the estimation | Yes, same method as proposed by authors in an earlier conference abstract | RCT data, external information on general population survival, conditional survival from cancer registry databases, and expert opinion. | • Survival in the control arm of the trial will remain less than survival in the matched general population cohort over the entire time horizon • 1-year conditional survival in the trial control arm will converge to that of a matched cancer cohort, five or six years after diagnosis. • HR changes over time as a smooth function with one turning point (expected to decrease initially and then increase) • HR assumed to "kick in" at 1 year, when treatment begins, and return to unity by 6 years  Bayesian method | WinBUGS, custom code |
| Cope *et al.* | 2019 | Expert opinion piecewise | • Elicit expert opinion for survival at 2, 3, 4, and 5 years via a SHELF-like tool • Synthesize estimates and combine with observed data using parametric models (accounting for uncertainty) • FP models used (due to the fact that there are no PLD for expert opinion) | No | PLD for index population + expert opinion (point estimates and uncertainty) | People alive after 5 years assumed to be long-term survivors  Bayesian method | JAGS and R |
| Lambert *et al.* | 2005 | Additive relative survival model + fractional polynomial relative survival model | Additive relative survival model: software for a GLM where covariates are reflected as additive rather than multiplicative. FP relative survival model: use FP model to reflect baseline excess hazard + potential time-varying covariates | Yes, considered to build on literature for relative survival | PLD for index population + demographic information to estimate general population survival | Additive model assumes covariates act additively on the excess hazard  Frequentist method | Stata |
| Lambert *et al.* | 2007 | NMCM | Fit a relative survival model assuming that a proportion of the cohort are cured, but instead of this being a mixture, assume the disease-related survival curve eventually asymptotes at this value | Yes, extends NMCM to relative survival | PLD for index population + demographic information to estimate general population survival | Proportion of patients 'cured' of the disease, who otherwise achieve the survival of the general population  Frequentist method | Stata |
| Lambert *et al.* | 2010 | Mixture of 2 Weibull models for uncured group in MCM/NMCM | Extends MCM/NMCM approach to consider a mix of two Weibull models for the uncured group. Otherwise, approach is same as per standard MCM/NMCM within a relative survival framework | Yes, extends MCM/ NMCM to relative survival | PLD for index population + demographic information to estimate general population survival | Proportion of patients 'cured' of the disease, who otherwise achieve the survival of the general population. Uncured group exhibits a complex hazard function, requiring a flexible two-component Weibull  Frequentist method | Stata |
| Andersson *et al.* | 2013 | Flexible parametric relative survival models | Method is an extension to the restricted cubic spline, described in detail by Royston and Parmar (2002).  To extend to the relative survival framework, consider S(t) = S*(t) x R(t), where S(t) = OS, S*(t) = expected survival, R(t) = relative survival. The overall hazards h(t) therefore estimated as the sum of the expected hazards (h*(t)) and the relative hazards (lambda(t)). Three alternative assumptions considered for extrapolation of the relative survival model:  • Assume log cumulative excess hazard beyond the last boundary knot follows the linear trend given by the estimated model parameters  • Assume statistical cure beyond the last boundary knot, by imposing constraints on spline parameters  • Assume constant excess hazard beyond the last boundary knot, by imposing constraints on spline parameters | Yes, extends spline model to relative survival | Two data sources required: first, PLD for index population; and second, all-cause mortality data to calculate the expected survival curve S*(t) | Requires the full all-cause survival curve of patients to be either known or estimated. Need to assume longer-term pattern of cause-specific hazards. In this study, three options were presented: (1) constant, (2) zero (i.e., "cure"), or (3) linear trend, akin to a "Weibull-like" tail  Frequentist method | Stata command stpm2 used to fit the flexible parametric models |
| Jakobsen *et al.* | 2019 | Flexible parametric relative survival models | Three different methods considered: • Nelson et al. relative survival model, which is linear on the log cumulative excess hazard scale after the last knot, • Andersson et al. relative survival model, which is constant on the log cumulative excess hazard scale after the last knot and thereby incorporates statistical cure • A flexible mixture cure model, which incorporates statistical cure, but is not restricted to a constant log cumulative excess hazard after the last knot | Yes, considers application of Andersson et al., (2013) and Nelson et al., (2007) + new method that addresses issues with Andersson et al., (2013) method | PLD for index population + life tables | Assumptions specific to each method RE: the extrapolated tail. Else, assumptions per standard relative survival approaches  Frequentist method | R, packages: rstpm2 and cuRe |
| Nelson *et al.* | 2008 | Age-based piecewise | Two-part model:  • First, a study model based on a Cox PH model fitted to study data  • Second, an extrapolation model using external data to describe hazard as a function of age, to then inform remainder of time horizon being modelled | Yes, references studies by authors where method used before, but this is the first methods paper | Two data sources required: first, PLD for index population; and second, an external data source to obtain an estimate of an age-based hazard function, controlling also for covariates (so ideally PLD) | Hazards stabilise after a given time point such that they can be represented as a function of patients current age + baseline covariates (not including age)  Frequentist method | Custom SAS code |
| Hawe *et al.* | 2014 | Combine model parameters | Three stages:  • Fit parametric models for the control arm in both studies with an indicator for study • Obtain treatment effect for active versus control by fitting models with covariates for both arm and study • Combine estimate of survival for control from Stage 1 with treatment effect from Stage 2 to obtain survival for active arm | No | Data 2 RCTs of the same control arms - ideally PLD for index population, but could digitise to obtain pseudo-PLD | Differences in survival can be described by covariate adjustment  Frequentist method | None listed |
| Hwang *et al.* | 2017 | Rolling extrapolation | Three stages:  • First, obtain a reference curve using a population with "known" hazard function. This is described as the "Monte Carlo method"  • Second, fit a spline model to the logit transform of the ratio of survival curves for both the index and the reference populations up to the end of follow-up  • Finally, use the regression + reference curve to estimate rest of survival curve | Yes, extension of the Monte Carlo method by the same lead author (only difference was instead of a spline model, this approach uses a simple linear regression) | Two data sources:  • PLD for index population  • Reference data for the general population (e.g., national statistics) from which counterfactual survival times (i.e., survival without the disease) may be simulated | Assumes that reference curve has a lower hazard of death versus trial group  Frequentist method | Links to R program developed by authors (iSQoL2): <http://sites.stat.sinica.edu.tw/isqol/> |
| Pennington *et al.* | 2018 | Summary data piecewise  External covariates | Two options. If only summary stats, can estimate a constant hazard for long-term. If ILD, fit joint models, and consider summary stats for long-term HR estimation. | Yes, uses elements of work by Demiris & Sharples + Benaglia *et al.*, but not in a Bayesian framework. Similar to Hawe *et al.* | Varies, but as a minimum PLD for index population + external data either at summary level, with potential PLD for the control arm at least | Assumes similarity of shape in hazard functions across populations. Differences expected to be explained via PH, log odds of event, or acceleration of time (model choice depending)  Frequentist method | Stata |
| Vickers | 2019 | Modified piecewise model  (multiple methods presented, but this method of primary relevant for this review) | 3-part model:  • First part informed by short-term data (target population)  • Second part by using adjusted external data until end of follow-up  • Final part informed by adjusted general population data. For active treatment, further adjustment by tapering the HR (i.e., treatment waning effect) | Yes, one method adapts the Guyot et al., (2017) approach, else methods use piecewise-style approaches | Data from 2 sources - a short-term (index population) study and long-term external study that is similar + general population survival estimates | Assumed similarity in populations, including constant AF describing differences between disease-specific sources, adjustment of HRs based on assumed time points  Frequentist method | R and JAGS. Packages used: eha, flexsurv, survPresmooth, MESS, ggplot2, and loo. |
| Soikkeli et al. | 2019 | Specification of a prior on the shape parameter | Fits a parametric model with an informative prior for the shape parameter informed via historical data | Yes, Bayesian alternative of the Hawe *et al.* method | Two data sets - one from a historical study for long-term shape parameter, and another to produce extrapolations for. Could perform method on pseudo-PLD or 'true' PLD for index population | Assumes PH/ constant AF if including treatment as a covariate. Otherwise, assumes 'similar' shape parameter/functional form of model across studies  Bayesian method | WinBUGS |
| Nelson et al. | 2007 | Relative survival spline model | Fits an additive hazards relative survival model, using a spline for disease-specific survival | Yes, extends spline model to relative survival | PLD for index population + background mortality estimates | Assumes hazards are additive  Frequentist method | Stata |
| Felizzi et al. | 2021 | Informed MCM | Fits a standard MCM but imposes the assumption that the cure fraction is fixed at a static value, obtained via some external information source | Yes, builds on a standard MCM | PLD for index population + background mortality estimates + relevant information for cure fraction and epidemiological information in target population | Can obtain an estimate of the cure fraction from an external source, and consider this 'fixed'  Frequentist method | R |
| van Oostrum et al. | 2021 | Internal additive hazards (i.e., relative survival)  External additive hazards  Convergence of hazards  Proportional hazards (PH)  (different background mortality methods) | Four different methods:  • Internal additive hazards (relative survival model)  • External additive hazards (add on background mortality, assuming negligible during follow-up)  • Converging hazards model (cap hazards by background mortality)  • PH model (estimate survival by applying a hazard ratio to a background mortality curve) | No | PLD for index population + background mortality estimates | Internal additive hazards model: Hazards related to disease and other causes are additive (not multiplicative)  External additive hazards model: In addition to the assumptions made for the internal additive hazards model, assumes general population hazards negligible during trial follow-up  Converging hazards model: Without adjustment, model would yield estimates of long-term hazards that fall below general population. This method assumes it is reasonable to ‘ignore’ implausible long-term extrapolation in favour of general population hazards. Indirectly, method also assumes ‘cure’ when switching to general population hazards  PH model: relationship between general population survival and the survival of the group of interest can be adequately represented via an HR  Frequentist method | R |
| Che *et al.* | 2021 | Blended survival model | Method involves fitting a model that comprises three piecewise periods:  • Initial period where survival follows that of the index group  • Mid-range period ('mixing area') where survival is considered a weighted average of the index and external groups  • Long-term period where survival is based on the external group only  The model is fitted making assumptions about the 'mixing area' | Yes, considered to take learnings from mixture-cure and poly-hazard models | PLD for index population + some form of external data such that a relevant time point (e.g., 10 years) and survival proportion (e.g., 20%) can be determined | Relies on several important parameter assumptions, including how 'quickly' the population follows the survival experience of the external cohort  Bayesian method | R (based on presentation, but not explicitly stated) |

**Key:** AAD, antiarrhythmic drug; AF, acceleration factor; AJCC, American Joint Committee on Cancer; FP, fractional polynomial; HR, hazard ratio; ICD, implantable cardioverter defibrillator; KM, Kaplan-Meier; NMCM, non-mixture-cure model; MCM, mixture-cure model; PH, proportional hazards; PLD, patient-level data; SEER, Surveillance, Epidemiology, and End Results Program; SHELF, Sheffield Elicitation Framework.

Table 7: Data extraction (Part 2 of 2)

| Article info | | Presentation and evaluation of method | | | Additional commentary | | |
| --- | --- | --- | --- | --- | --- | --- | --- |
| Author(s) | Year | Application | Validation | Code | Advantages (from author) | Limitations (from author) | Any other comments |
| Larkin *et al.* | 2015 | Melanoma - trial data + registry data + life tables. Used in context of explaining approach taken in HTA | Validated extrapolations by speaking to clinical experts. Also compared projections to published long-term survival data for ipilimumab from Prieto *et al.* (56 patients from a clinical trial of previously treated patients) and the recently published pooled analysis of 1,861 patients over all lines of therapy reported by Schadendorf *et al.*, which was not available at the time of analysis) | No | More realistic extrapolation based on evident plateau + aligned with clinical opinion versus conventional parametric approaches. Reduced mean absolute error in predictions | None described |  |
| Barlev *et al.* | 2017 | Adults with leukaemia - trial data + observational study + life tables. | Repeated analysis with a later data cut - commented that extrapolations were consistent | No | No other approaches discussed. | Limitations primarily related to use of historical data, including assumption of no additional effects of blinatumomab beyond two years, potential changes in treatment practices over time in the clinical vs historical populations and differences in transplant realisation rates |  |
| Fisher *et al.* | 2017 | IPF - trial data + observational study | Mentioned not possible to validate as no other external data sources available | No | No other options mentioned, but implied that parametric models fitted to 1 year of data unlikely to do well | Potential biases with Inova being a registry. Definition of best supportive care may differ between trial + registry and may have changed over time. Registry KM prior to 52 weeks not same as trial | Included an element of adjusting data to ‘match’ the index population, but is otherwise a standard piecewise model |
| Demiris & Sharples | 2006 | Authors present an example concerning ICD therapy. Data come from a cohort study, UK life tables, and a meta-analysis of three RCTs | No specific validation approaches presented, but authors do compare the proposed methods to a standard Weibull and compare fits. | No. Code is cited but hosted on webpage that no longer exists. Author contacted but no response | Method performs well, compared to a standard Weibull model. Bayesian methods are 'convenient' when prior and likelihood information is to be considered | The gamma process adopted for modelling the baseline hazard considered a basic approach but stated by authors to appear to work very well for the example presented. Authors acknowledge that other more sophisticated approaches exist in the literature which might be useful alternatives should more complicated prior structures for the baseline hazard be considered |  |
| Benaglia *et al.* | 2015 | Applied to a cohort of patients that received implantable cardioverter defibrillators. | Simulation study. Poly-hazard model was compared to two "incorrectly-specified" models. The incorrectly specified models did not account for the cause-specific effect of an intervention on the hazard of death but assume that the study patients have a proportionally increased hazard for overall survival compared to the general population. Outside the context of the simulation study, the authors fit two different poly-hazard models (poly-Weibull and poly-Gompertz) to the implantable cardioverter defibrillators data. The models are compared in terms of mean overall life-years, and their "fit" to the KM estimate and an empirical hazard plot (methods to produce this unclear) | Yes, provided as an appendix within the article ("Appendix A: WinBUGS model code") | Able to address potential bias where simply assuming proportional hazards for all-cause mortality when estimating expected lifetime survival. | Need to consider sensitivity analyses, and careful and consistent categorisation of causes of death in survival data, as results can be highly sensitive to assumed reference population causes of death. | Paper notes that the authors have focused on Weibull and Gompertz, which have the PH property which makes estimation and interpretation slightly simpler (and allows for use of published HRs if relevant). |
| Guyot *et al.* | 2017 | RCT conducted in head and neck cancer | Compared to standard models with no external information included | Yes, included in an appendix | Allows for fitting of a single, consistent model that acknowledges all data sources | Issues of generalisability, potential age of studies, method would be difficult to use where data are limited | Computationally challenging approach, owing to the variety of inputs required. This approach attempts to use a broad range of evidence sources to inform model estimation |
| Cope *et al.* | 2019 | Trial in leukaemia (young adults, curative potential) | Validated using a later data cut | No | Leads to an increase in the precision of survival estimates versus trial-data based estimates alone | Limited ability to validate approach given small sample + censoring | Use of FP models in this context is non-standard and computationally complex |
| Lambert *et al.* | 2005 | Large breast cancer cohort (>100,000) to illustrate different ways of estimating relative survival | None reported | No. Code is cited, but hosted on webpage that no longer exists | Additive hazards: Ease of interpretation, can be used to capture zero or potential negative covariates (e.g., covariates meaning that after cure patients have better survival than general population). FP models: a wide range of plausible curves can be explored, use fewer parameters than splines/ piecewise models | None explicitly described | Different ways of specifying a relative survival model. FP approach relies on a lot of data |
| Lambert *et al.* | 2007 | Illustrative examples presented for patients with ovarian cancer | Compared to mixture-cure models | No | The NMCM has a PH model as a special case for the whole population (the MCM does not have this property) | None described |  |
| Lambert *et al.* | 2010 | Illustrative examples presented for patients with colon cancer | None reported | Yes, link to Stata package included (Lambert, 2007, Stata journal) | Increased flexibility in the shape of the excess hazard or relative survival function versus conventional MCMs/NMCMs | None described |  |
| Andersson *et al.* | 2013 | Applied to data from the Swedish Cancer Registry. Data from four different cancers. | Method evaluated and presented using Swedish cancer registry data (but no validation of these extrapolations separately) | No, but references updates made to the stpm2 command in Stata. | Using a relative survival approach gives better estimates of the full all-cause survival function. Other approaches when applied to grouped data have assumed either cure or a long-term fixed hazard of death. However, in this approach, the extrapolation can also be carried out by extrapolating the linear trend given by the estimated model parameters. Estimation can be carried out on PLD and not only on grouped data | Potential problems include sensitivity to the number of knots uses, and the amount of follow-up needed for the extrapolation to perform well. Authors used naive assumption that the population mortality rates stay the same from 1980 onwards. Extrapolations could be poor if background mortality rates change substantially over time. Approach has not been evaluated on small datasets or data from clinical trials or cost-effectiveness studies (but could be useful in these kind of studies) | Paper focused predominantly on estimating the loss in expectation of life due to cancer, as opposed to the all-cause mortality curve itself. However, authors note that this method can be used for estimating the survival curve (but the metric of interest to the study may be considered useful especially within the context of defining the burden of disease and comparing cancers) |
| Jakobsen *et al.* | 2019 | Danish lymphoma registry | Simulation study | No | None described | None described |  |
| Nelson *et al.* | 2008 | Patients that have had an MI | Some later 5-year data reported | Yes, included in an appendix | Ability to adjust for covariates. Does not assume a functional form because Cox PH rather than Weibull, Gompertz etc. Makes predominantly "weak" assumptions. Can be implemented in standard software (e.g., SAS). Use of age metric makes efficient use of available data | Complex methodology. Not applicable to all situations (e.g., small sample sizes) | Relies on a lot of data and complex to implement (even though code is available) |
| Hawe *et al.* | 2014 | Two RCTs in CLL | None reported | No | Can be used to make use of external data when absolute outcomes differ | Assumes PH/ constant AF (depending on model fitted) between the study arms Choice of model could influence extrapolations markedly Difficult to validate given lack of separate long-term data | Potentially helpful within the context of having access to the two studies with the same control arm, but this is usually not the case in HTA |
| Hwang *et al.* | 2017 | Simulation study + illustrative example | Simulation study + compared to a standard Weibull model | Yes, in the form of the iSQoL2 package | Does not require excess hazards to be constant over time | Logit W(t) values may be unstable if small n + limited follow up, which could cause issues in the rolling extrapolation algorithm | Modification to the Monte Carlo method by the same authors |
| Pennington *et al.* | 2018 | Data set from the National Joint Registry, about knee replacements | Compared estimates relative to "best possible model" using all available data | Yes, included in an appendix | Could help improve estimates of cost-effectiveness (assuming estimates without external data are pessimistic) | Risk of selection bias when using observational data. Some specific assumptions made on relationship between curves, which may not be possible to verify. |  |
| Vickers | 2019 | Semi-simulated data from patients with NSCLC. External data from a published paper + general population data | Compared extrapolations to external data, but no formal validation presented | No. Some code included in an appendix, but not the full code for all reported analyses | External data needed to produce accurate extrapolations in presence of a 'large' treatment effect | Relies on similarity of external data. Need to assume time points to taper HR | A series of quite complex adjustments to three data sources to ultimately give a piecewise-style model. Best performing method identified by the author was the Guyot *et al.*, (2017) method |
| Soikkeli et al. | 2019 | Newly diagnosed MM (RCT population) | Extrapolations validated against a later data cut from the same trial | Yes, included as supplementary material | Improved upon extrapolations without specification of a prior in the example presented | Not suitable for situations where a constant treatment effect cannot be specified (if capturing multiple treatment groups). Limited flexibility of the standard parametric models considered |  |
| Nelson et al. | 2007 | Real-world population with coronary heart disease | Compared with 'standard relative survival analyses' | No, but 'Stata program available from the authors on request.' | More flexible approach to model excess hazard, avoids needs to split timescale (as would be needed in a piecewise approach) | Number of knots can be influential on results |  |
| Felizzi et al. | 2021 | Illustrated with an example in advanced melanoma | None presented | Yes, provided a link to a GitHub repository | Can address limited data available to robustly estimate an MCM via an uninformed approach | Requires ILD, cure fraction may be uncertain and not reflected in the model, and potential differences in trial versus background mortality populations |  |
| van Oostrum et al. | 2021 | Newly diagnosed MM (RCT population) + breast cancer (another RCT) | Extrapolations validated against a later data cut from the same trial | No | General population mortality data are routinely available in the UK. Transparent and intuitive application of general population data to inform extrapolations | None explicitly highlighted for the approach in general. For the PH model, the ability to justify PH holds is a limitation. The converging hazards require assumption of cure which may not hold, and if it does hold cure-based models may be preferred. External additive hazards requires general population hazards to be negligible during follow-up | Range of background mortality methods, though authors comment that internal additive hazards seems to perform best |
| Che *et al.* | 2021 | RCT in CLL | None presented | No | • Overcomes the overly optimistic constant treatment effect • Sufficient flexibility with various plausible scenarios • Less concerned about model selection | • Elicited belief: probably a weak form of evidence, need long-term data • Does not (yet) account for other kind of external knowledge | Similar to a piecewise model, except there is a gradual movement away from data for the index population to the external population (as opposed to an immediate change), and the model is fitted in one consistent framework |

**Key:** AF, acceleration factor; CLL, chronic lymphocytic leukaemia; FP, fractional polynomial; HR, hazard ratio; HTA, health technology assessment; ICD, implantable cardioverter defibrillator; IPF, idiopathic pulmonary fibrosis; KM, Kaplan-Meier; NMCM, non-mixture-cure model; NSCLC, non-small-cell lung cancer; MCM, mixture-cure model; MI, myocardial infarction; MM, multiple myeloma; PH, proportional hazards; PLD, patient-level data; RCT, randomised controlled trial.

**References**

1. Hooijmans CR, Tillema A, Leenaars M, Ritskes-Hoitinga M. Enhancing search efficiency by means of a search filter for finding all studies on animal experimentation in PubMed. Lab Anim. 2010 Jul;44(3):170–5.

2. Larkin J, Hatswell AJ, Nathan P, Lebmeier M, Lee D. The Predicted Impact of Ipilimumab Usage on Survival in Previously Treated Advanced or Metastatic Melanoma in the UK. PLoS One. 2015;10(12):e0145524.

3. Barlev A, Lin VW, Katz A, Hu K, Cong Z, Barber B. Estimating Long-Term Survival of Adults with Philadelphia Chromosome-Negative Relapsed/Refractory B-Precursor Acute Lymphoblastic Leukemia Treated with Blinatumomab Using Historical Data. Adv Ther. 2017 Jan;34(1):148–55.

4. Fisher M, Nathan SD, Hill C, Marshall J, Dejonckheere F, Thuresson PO, et al. Predicting Life Expectancy for Pirfenidone in Idiopathic Pulmonary Fibrosis. J Manag Care Spec Pharm. 2017 Mar;23(3-b Suppl):S17–24.

5. Nelson CL, Sun JL, Tsiatis AA, Mark DB. Empirical estimation of life expectancy from large clinical trials: use of left-truncated, right-censored survival analysis methodology. Stat Med. 2008 Nov 20;27(26):5525–55.

6. Pennington M, Grieve R, der Meulen JV, Hawkins N. Value of External Data in the Extrapolation of Survival Data: A Study Using the NJR Data Set. Value Health. 2018 Jul;21(7):822–9.

7. Che Z, Baio G, Paly VF. At First I Was Afraid, I Was Petrified... Issues and Possible Solutions to the Problems of Extrapolating Survival Curves from Limited Trial Data [Internet]. ISPOR | International Society For Pharmacoeconomics and Outcomes Research. 2021 [cited 2022 Jul 15]. Available from: https://www.ispor.org/heor-resources/presentations-database/presentation/euro2021-3402/13243

8. Soikkeli F, Hashim M, Ouwens M, Postma M, Heeg B. Extrapolating Survival Data Using Historical Trial–Based a Priori Distributions. Value in Health. 2019 Sep 1;22(9):1012–7.

9. Vickers A. An Evaluation of Survival Curve Extrapolation Techniques Using Long-Term Observational Cancer Data. Med Decis Making. 2019 Nov;39(8):926–38.

10. Cope S, Ayers D, Zhang J, Batt K, Jansen JP. Integrating expert opinion with clinical trial data to extrapolate long-term survival: a case study of CAR-T therapy for children and young adults with relapsed or refractory acute lymphoblastic leukemia. BMC Med Res Methodol. 2019 02;19(1):182.

11. Guyot P, Ades AE, Beasley M, Lueza B, Pignon JP, Welton NJ. Extrapolation of Survival Curves from Cancer Trials Using External Information. Med Decis Making. 2017;37(4):353–66.

12. Benaglia T, Jackson CH, Sharples LD. Survival extrapolation in the presence of cause specific hazards. Statistics in Medicine. 2015;34(5):796–811.

13. Demiris N, Sharples LD. Bayesian evidence synthesis to extrapolate survival estimates in cost-effectiveness studies. Stat Med. 2006 Jun 15;25(11):1960–75.

14. Lambert PC, Smith LK, Jones DR, Botha JL. Additive and multiplicative covariate regression models for relative survival incorporating fractional polynomials for time-dependent effects. Stat Med. 2005 Dec 30;24(24):3871–85.

15. Lambert PC, Thompson JR, Weston CL, Dickman PW. Estimating and modeling the cure fraction in population-based cancer survival analysis. Biostatistics. 2007 Jul;8(3):576–94.

16. Lambert PC, Dickman PW, Weston CL, Thompson JR. Estimating the cure fraction in population-based cancer studies by using finite mixture models. Journal of the Royal Statistical Society: Series C (Applied Statistics). 2010;59(1):35–55.

17. Nelson CP, Lambert PC, Squire IB, Jones DR. Flexible parametric models for relative survival, with application in coronary heart disease. Stat Med. 2007 Dec 30;26(30):5486–98.

18. Andersson TML, Dickman PW, Eloranta S, Lambe M, Lambert PC. Estimating the loss in expectation of life due to cancer using flexible parametric survival models. Stat Med. 2013 Dec 30;32(30):5286–300.

19. Jakobsen LH, Andersson TML, Biccler JL, El-Galaly TC, Bøgsted M. Estimating the loss of lifetime function using flexible parametric relative survival models. BMC Med Res Methodol. 2019 Jan 28;19(1):23.

20. Hwang JS, Hu TH, Lee LJH, Wang JD. Estimating lifetime medical costs from censored claims data. Health Econ. 2017 Dec;26(12):e332–44.

21. van Oostrum I, Ouwens M, Remiro-Azócar A, Baio G, Postma MJ, Buskens E, et al. Comparison of Parametric Survival Extrapolation Approaches Incorporating General Population Mortality for Adequate Health Technology Assessment of New Oncology Drugs. Value Health. 2021 Sep;24(9):1294–301.

22. Felizzi F, Paracha N, Pöhlmann J, Ray J. Mixture Cure Models in Oncology: A Tutorial and Practical Guidance. Pharmacoecon Open. 2021 Jun;5(2):143–55.

23. Hawe E, Pearson I, Wolowacz S, Haiderali A. Use Of External Data To Guide Long-Term Survival Extrapolations Of Trial Data For Chronic Lymphocytic Leukemia. Value Health. 2014 Nov;17(7):A563.
